# Non-invasive micro-test technology and applications

**DOI:** 10.52601/bpr.2024.240009

**Published:** 2025-04-30

**Authors:** Kai Sun, Yunqi Liu, Yanshu Pan, Dongwei Di, Jianfang Li, Feiyun Xu, Li Li, Yoshiharu Mimata, Yingying Chen, Lixia Xie, Siqi Wang, Wenqian Qi, Yan Tang, Huachun Sheng, Bing Wang, Ruixue Sun, Dingquan Tan, Daohong Fu, Ye Yin, Ao Xue, Yichao Shi, Wenjing Shao, Lei Gong, Zhijian Jiang, Wei Zhang, Qiangsheng Wu, Yaosheng Wang, Minglin Lang, Wenxiu Ye, Weifeng Xu, Shuhe Wei, Weiming Shi, Yue Jeff Xu

**Affiliations:** 1 College of Life Sciences, Nanjing Normal University, Nanjing 210046, China; 2 Zhongguancun Xuyue NMT Industrial Alliance, Beijing 100080, China; 3 NMT International Alliance, Amherst, Massachusetts 01002, USA; 4 College of Traditional Chinese Medicine, Beijing University of Chinese Medicine, Beijing 102488, China; 5 State Key Laboratory of Soil and Sustainable Agriculture, Institute of Soil Science, Chinese Academy of Sciences, Nanjing 210008, China; 6 College of Biological Science, China Agricultural University, Beijing 100193, China; 7 Center for Plant Water-use and Nutrition Regulation and College of Resources and Environment, Joint International Research Laboratory of Water and Nutrient in Crop, Fujian Agriculture and Forestry University, Fuzhou 350002, China; 8 Institute of Agricultural Resources and Environment, Tianjin Academy of Agricultural Sciences, Tianjin 300380, China; 9 Peking University Institute of Advanced Agricultural Sciences, Shandong Laboratory of Advanced Agricultural Sciences at Weifang, Weifang 261000, Shandong, China; 10 Guangxi Forestry Research Institute, Nanning 530002, China; 11 Institute of Wetland Agriculture and Ecology, Shandong Academy of Agricultural Science, Jinan 250100, China; 12 Key Laboratory of Wastewater Treatment Technology of Liaoning Province, Academy of Environmental and Chemical Engineering, Shenyang Ligong University, Shenyang 110159, China; 13 Key Laboratory of Tropical Marine Bio-resources and Ecology, South China Sea Institute of Oceanology, Chinese Academy of Sciences, Guangzhou 510301, China; 14 Technical Institute of Physics and Chemistry, CAS, Beijing 100190, China; 15 Institute of Qinghai-Tibetan Plateau, Southwest Minzu University, Chengdu 610225, China; 16 Molecular Genetics Key Laboratory of China Tobacco, Guizhou Academy of Tobacco Science, Guiyang 550081, China; 17 College of Agriculture and Forestry, Hebei North University, Zhangjiakou 075000, Hebei, China; 18 Smart Health Institute, Chongqing Vocational College of Media, Chongqing 402560, China; 19 Institute of Biology, Humboldt University of Berlin, Berlin 10099, Germany; 20 College of Horticulture, Qingdao Agricultural University, Qingdao 266109, Shandong, China; 21 College of Pharmacy, Heilongjiang University of Chinese Medicine, Harbin 150040, China; 22 Department of Gastroenterology, Aerospace Center Hospital, Peking University Aerospace School of Clinical Medicine, Beijing 100049, China; 23 CAS Center for Excellence in Biotic Interactions, College of Life Science, University of Chinese Academy of Sciences, Beijing 100049, China; 24 State Key Laboratory of Grassland Agro-ecosystems, School of Life Sciences, Lanzhou 730000, China; 25 College of Horticulture and Gardening, Yangtze University, Jingzhou 434025, Hubei, China; 26 Institute of Environment and Sustainable Development in Agriculture, Chinese Academy of Agricultural Sciences, Beijing 100081, China; 27 Key Laboratory of Pollution Ecology and Environment Engineering, Institute of Applied Ecology, Shenyang 110016, China; 28 Xuyue (Beijing) Sci. & Tech. Co., Ltd., Beijing 100080, China; 29 YoungerUSA LLC, Amherst, Massachusetts 01002, USA

**Keywords:** Non-invasive micro-test technology, Transmembrane transport, Ionic homeostasis, H^+^-ATPase, Calcium signature, Physiology

## Abstract

Non-invasive micro-test technology (NMT) reveals dynamic ionic/molecular concentration gradients by measuring fluxes of ions and small molecules in liquid media in 1D, 2D or 3D fashions with sensitivity up to pico- (10^−12^) or femto- (10^−15^) moles per cm^2^ per second. NMT has been applied to study metabolism, signal transduction, genes and/or proteins physiological functions related to transmembrane ionic/molecular activities with live samples under normal conditions or stress. Data on ion and/or molecule homeostasis (IMH) by NMT in biomedical sciences, plant and crop sciences, environmental sciences, marine and space biology as well as traditional Chinese medicine are reviewed.

## INTRODUCTION

Ion and/or molecule homeostasis (IMH) is a fundamental biological phenomenon essential for all living organisms (Ahmadi *et al*. 2023; Huang *et al*. 2022; Song *et al*. 2024; Sun *et al*. 2009a, 2009b, 2010; Xu 2023; Xu *et al*. 2022; Yang *et al*. 2010). Pathology of humans, animals, crops, insects and microbes is accompanied by abnormal IMHs. Excessive H^+^ accumulations, for instance, are the result of lactate formation even in the presence of O_2_ in tumor cells (Martinez-Outschoorn *et* *al*. 2011; Semenza 2007; Seyfried and Shelton 2010; Warburg 1956; Warburg *et al*. 1927). It is also well known that a plant will not grow properly if the supply of NH_4_^+^, NO_3_^−^, PO_4_^3−^, K^+^ are out of balance (Guan *et al*. 2024; Martínez-Ballesta *et al*. 2020; Tyler 2017; Zhou *et al*. 2019).

However, it has been a challenge for biologists to fully comprehend IMHs due to: (1) ionic and molecular information related to IMH have to be collected with live samples; (2) multiple ions/molecules are preferably measured simultaneously in order to construct the network among relevant ion(s)/molecule(s); (3) ionic/molecular data in different levels, *i*.*e*. cell, tissue to organ, has to be collected ; and (4) since biological materials are 3-dimensional per se, 1D or 2D form of data is no longer accurate to reflect the 3D world (Kunkel *et al*. 2001, 2006; Wan *et al*. 2011; Xu *et al*. 2006; Yang *et al*. 2012).

Non-invasive micro-test technology (NMT) is a collection of techniques. Initial NMT hardware was developed based on the Vibration Probe (VP) Technique invented by Dr. Lionel Jaffe at Marine Biological Laboratory in the early 1990s (Degenhardt *et al*. 1998; Kochian *et al*. 1992; Kuhtreiber and Jaffe 1990; Smith 1995). VP was primarily focused on solving the problems of voltage signal drifts and instrumental background noise (Kunkel *et al*. 2006; McLamore and Porterfield 2011), while NMT went further emphasizing: (1) developing more ionic and molecular micro-sensors; (2) simultaneous multiple ion/ion and/or ion/molecule measurements; (3) 3D-flux data collection and visualization and (4) automation of NMT to be more efficient to collect flux data (Kunkel *et al*. 2006; Xu *et al*. 2006; Yin *et al*. 2006). The term Non-invasive Micro-test Technology, initially proposed by Yue Jeff Xu, is now widely accepted (Xu 2023).

This review will describe NMT about: (1) working principles based on the 2-point measurement scheme (2PMS); (2) spatial relationships among samples, NMT sensors and ionic/molecular gradients from physical and chemical perspectives; and (3) ionic/molecular fluxes in relation to their gradients.

Comparisons will be made between NMT with other ion/molecule detection techniques, such as patch clamp and ion-selective microelectrodes.

NMT is now widely used in plant and crop sciences (Liu *et al*. 2023), animal science, microbiology, environmental science, and traditional Chinese medicine, as outlined below.

## NON-INVASIVE MICRO-TEST TECHNOLOGY (NMT)

### Principles

#### Two-points measurement scheme

**Figure 1:**
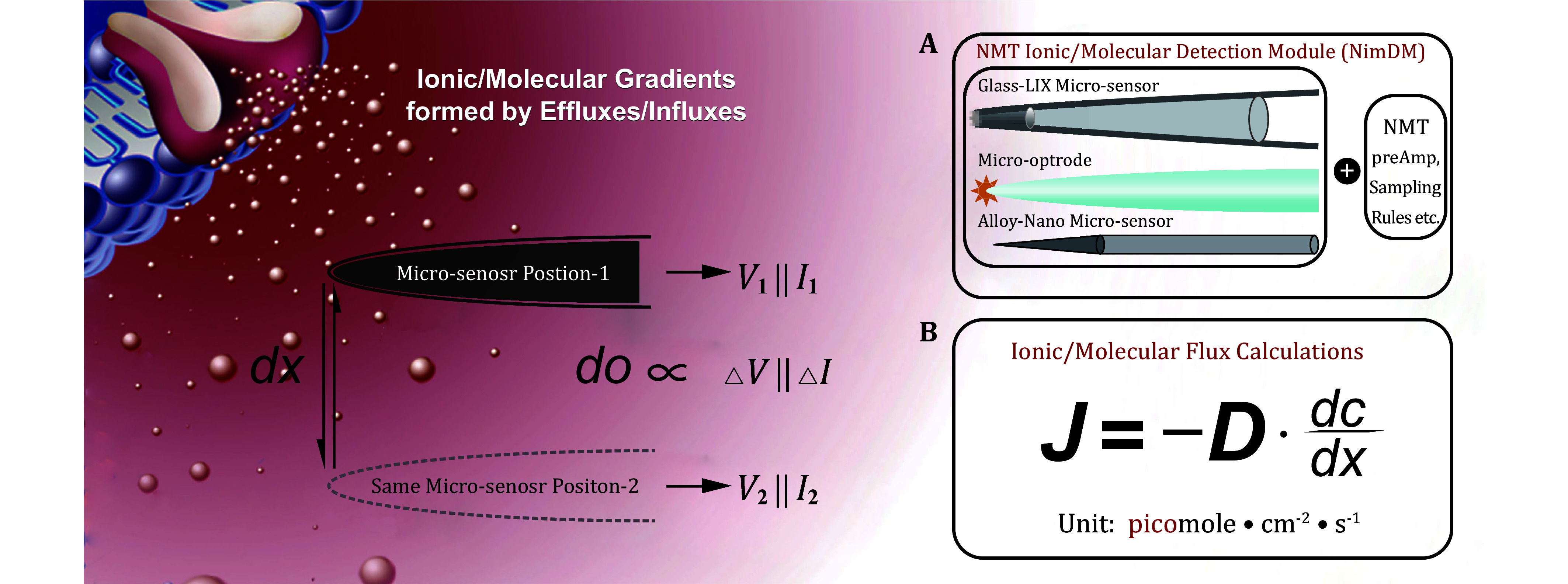
Working Principle of NMT. Diagram of NMT working theory. A NMT ionic/molecular micro-sensor undergoes two-point measurement at a known distance *dx* in the concentration gradient of the ions/molecules obtaining voltages *V*_1_||*I*_1_ and *V*_2_||*I*_2_ respectively. The concentration difference *dc* between the two points is proportional to _Δ_*V*||_Δ_*I* derived from *V*_1_||*I*_1_ and *V*_2_||*I*_2_. **A** Fluorescent dyes/optical fibers, nano-carbon filaments, enzyme electrodes, fabricated metals/alloys, *etc*. can all be used to detect ions/molecules. **B** Fick's first law of diffusion is adopted to calculate ionic/molecular fluxes, where *D* is the diffusion coefficient of ions/molecules (cm^2^/s)

Ionic/molecular fluxes are detectable when ions/molecules move from high concentrations to low concentrations. The concentration differences then result in ionic/molecular gradients (Fig. 1). In live biological samples, transmembrane activities of ions/molecules result in ionic/molecular gradients via their effluxes and/or influxes (Kunkel *et al*. 2001). In order to measure these ions/molecules selectively or specifically in a quantitative way, various sensors made from glass, optical fiber, and platinum-iridium alloy have been developed. These sensors’ fabrications are based on different theories/mechanisms, such as ionophores, enzymatic reactions, electrochemistry, and fluorescent dyes (McLamore and Porterfield 2011).

Due to the special demands of temporal and spatial resolutions, conventional ionic/molecular sensors usually have to be modified to fit NMT hardware configuration. Therefore, NMT ionic/molecular micro-sensors are designated to differentiate themselves from those traditional micro-sensors.

#### 1D, 2D, 3D and 6D measurements

NMT flux data in 1D, 2D and 3D are essential to (1) validate results from molecular biology, biochemistry, microscopy, and omics experiments; (2) provide clues for research on new genes, and new mechanisms; (3) for network map connecting among genes, proteins, metabolites.

**Figure 2:**
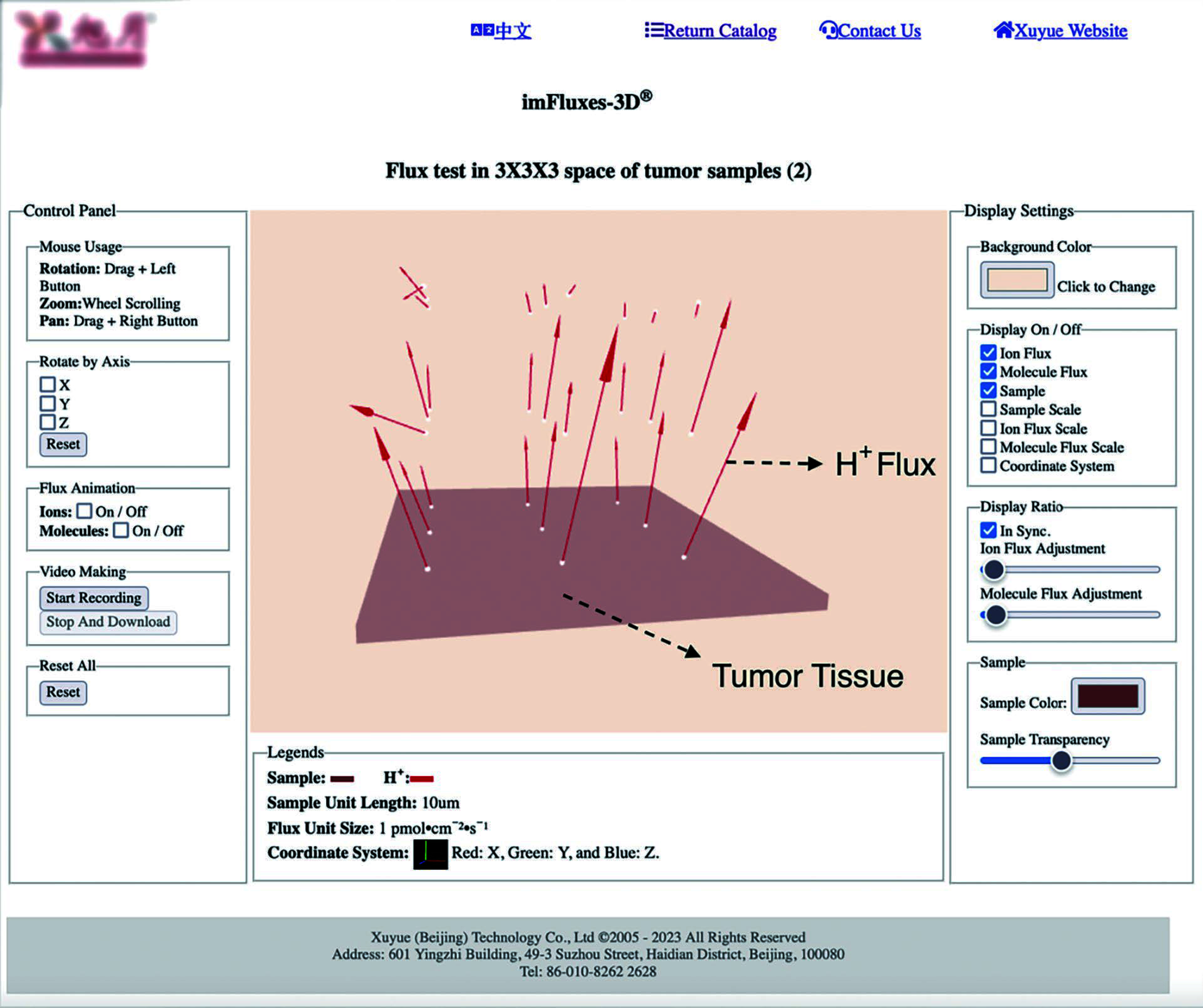
3D animation. Screenshot of imFluxes3D^®^ online version. Top: navigation area and title of current 3D file displayed; Left: Control Panel; Middle: area for displaying the 3D animation of samples, ionic/molecular fluxes and legends. It will be updated in real time as the user adjusts the parameters in both “Control Panel” and “Display Settings”. Bottom: legends to the 3D animation above is displayed here; Right: Display Settings

An online interactive 3D animation application has been developed to not only visualize 3D flux data but also for additional features, such as zooming, spinning, panning, movie making and coloring (Fig. 2) (Wang *et al*. 2023b).

A recent NMT hardware design allows both NMT micro-sensor and the tested sample to move in their own 3D system independently, which is even more powerful with a versatile 6D NMT system (Xu 2023).

### Limitations of NMT

The types of NMT sensors and their temporal and spatial resolutions are limiting factors of NMT.

#### NMT Micro-sensors

Knowing exactly which ions/molecules are measured is very straightforward, but adding new members to the current NMT sensor’s list is not an easy task. For example, some breakthroughs have been made to the most desired Fe^2+^/^3+^ and PO_4_^3−^ sensors recently after years of endeavors and efforts. The reasons that this progress has been slow are (1) only a few scientists are working in the field; (2) due to the inter-disciplinary nature of NMT sensor’s development; (3) unwillingness to develop new NMT sensors for the lack of commercial incentives.

#### Temporal resolution

NMT’s temporal resolution is mainly determined by its responding time. When obtaining reliable and stable readings, the NMT sensor requires responding time in a few seconds range versus dozens of seconds or even minutes of conventional microsensors.

**Figure 3:**
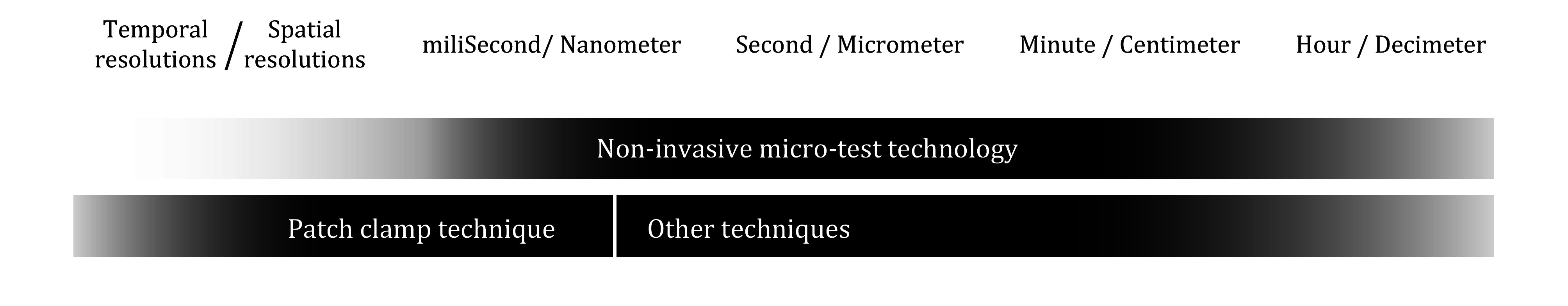
NMT temporal and spatial resolutions. Comparisons between NMT and other techniques. NMT resides in the range covering the gap between patch clamp and other techniques

Although temporal resolution may be a limiting factor for NMT to go below seconds, biologists are taking advantage of its relative long-term detection capability of up to hours or even days (Fig. 3).

### Comparative advantages over other techniques

#### NMT vs. Micro-electrodes

**Figure 4:**
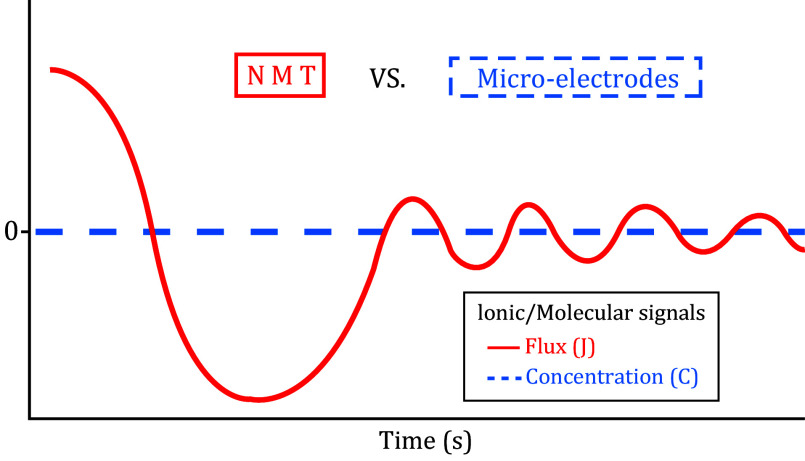
NMT versus micro-electrodes. Comparison between NMT and micro-electrodes. Under identical experimental conditions, NMT can pick up reliable and quantifiable ionic/molecular flux signals while micro-electrodes do not

NMT is different from traditional Ion Selective and/or molecular specific Micro-electrodes. On one hand, it is true to a certain extent because they both inform about the ions and/or molecules, on the other hand, much difference exists upon how much information each technique can reveal (Fig. 4).

#### NMT vs. Patch Clamp

E. Neher and B. Sakmann were awarded the Nobel Prize in 1991, after they invented the patch clamp technique to measure ionic currents in the plasma membrane of living cells (Xu and Qiu 1993).

One of the major breakthroughs with patch clamp is the successful GigaΩ seal between the polished glass micro-pipette and the plasma membrane. Yet, it also brings in significant mechanical impacts or even damages to the cells, which makes the mechanical stimulations unavoidable throughout every patch clamp experiment (Yang *et al*. 2012).

Therefore, a non-invasive way of detecting ions across cell membranes is very attractive. Especially for plant biologists to study salt or water processes, there is no need to enzymatically peel away the cell walls of plant cells which perturb the properties of cell membranes significantly (Xu 2023).

**Figure 5:**
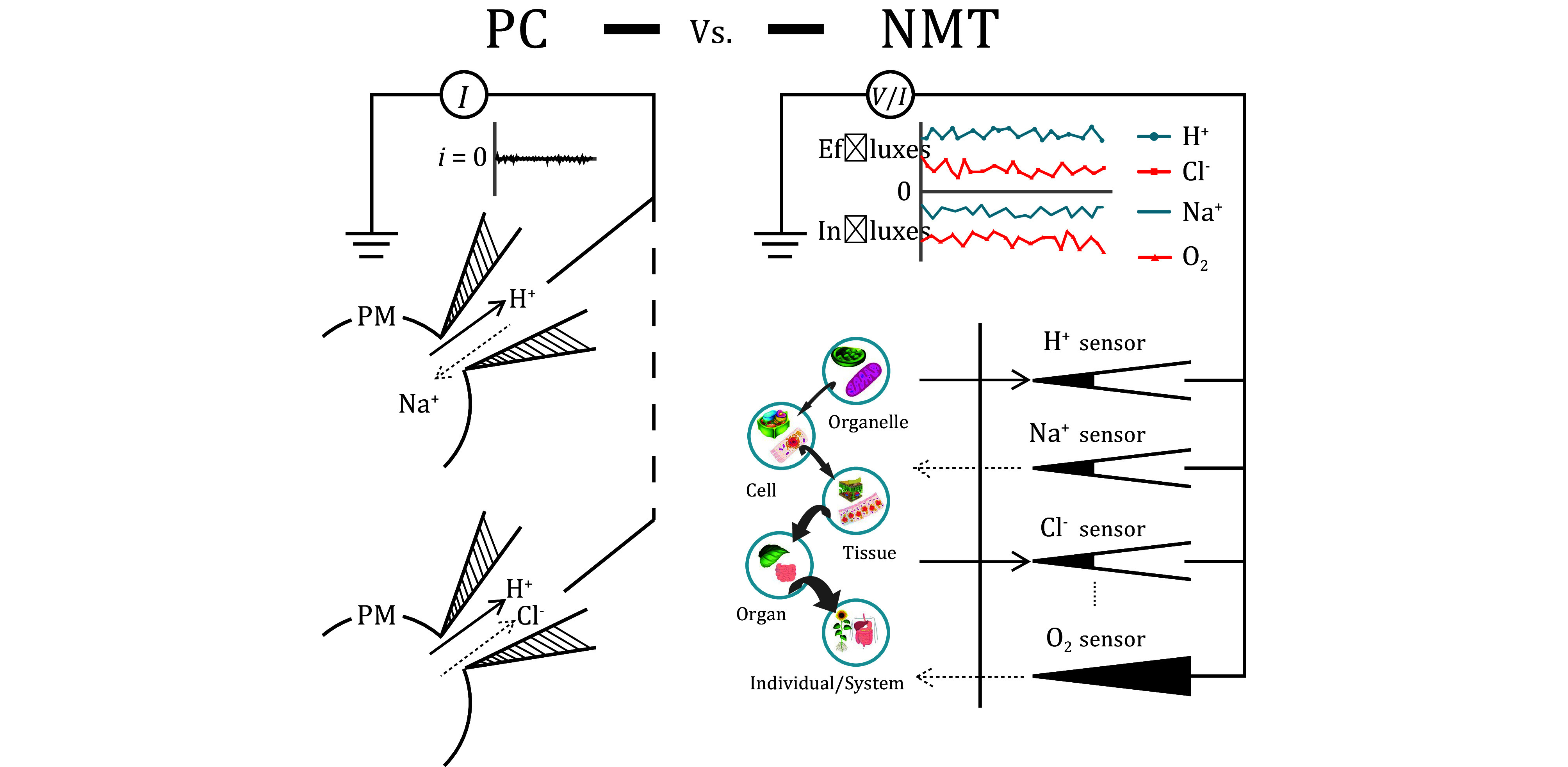
NMT versus Patch Clamp. Comparison between NMT and Patch Clamp technique. Left panel: there are no currents when two identical electrically charged ions move in the opposite direction, as well as two opposite electrically charged ions moving in the same direction. Right panel: regardless of the sizes of the samples and their electrical charges, as long as there are specific ionic/molecular sensors available, NMT can pick up all the ionic and molecular signals. H^+^, Na^+^, Cl^−^ and O_2_ sensors are used to illustrate not only the different types of NMT sensors used simultaneously with different samples, but also effluxes and/or influxes of multiple ions/molecules detected at the same time in one NMT experiment. In the following, we will outline some recent progress made in different research areas in biology and medicine with the assistance of the NMT technology

NMT can detect ion(s) movement(s) even under electrical neutral conditions (Fig. 5).

A valuable by-product of no-touching measurements of NMT is that it can measure a wide range of live samples in different sizes, from small intact organisms, isolated organs, tissues, cell layers, and single cells to aggregated organelles or microbes.

Detection of multi-ions/molecules simultaneously is another unique feature of NMT, which allows scientists to study the inter-relationships not only among ions but also among ions and molecules potentially paving the way to understand vital physiological functions of life (Kunkel *et al*. 2006; Xu *et al*. 2006).

However, patch clamps have good temporal and spatial resolutions (Fig. 3), which allow scientists to take advantage of both techniques to tackle tough scientific questions (Chen *et al*. 2007; Shabala *et al*. 2006; Smith *et al*. 1999).

## NMT APPLICATIONS

### Medicine and healthy

#### Disease treatments

Glioma is a common brain tumor with high morbidity and mortality. Recent studies have shown that photodynamic therapy (PDT) can be used to kill the glioma cells due to increased extracellular glutamate concentrations in C6 gliomas and upregulated glutamate receptor AMPA expression. However, how PDT affects the related transportations of Ca^2+^ and K^+^ across the cell membranes remains elusive. Researchers have demonstrated with NMT that significant Ca^2+^ uptake and K^+^ loss occurred after PDT treatment resulting in the death of C6 glioma cells (Hu *et al*. 2014). Antagonist CNQX eliminated the changes of Ca^2+^ and K^+^ therefore direct evidence was provided for the involvement of changes in Ca^2+^ and K^+^ transportations in PDT interventions (Hu *et al*. 2014). To date, NMT has been successfully used to correlate both O_2_ and Ca^2+^ fluxes to the early signals of PDT-induced apoptosis as well as the intracellular ROS (Song *et al*. 2015). Intracellular pH (pHi) and extracellular pH (pHe) are important parameters for maintaining various physiological functions. The drug resistance study method (DRSM) was established using non-invasive micro-test technology (NMT), which can be used to study the correlations among organs, tissues, extracellular ions, molecular activities and anti-cancer drug resistance (Ma *et al*. 2012). The study suggested the correlation between extracellular H^+^ activity and anti-cancer drug resistance by using ADR (doxorubicin) with both sensitive and resistant strains of MCF-7 cells (Song *et al*. 2008).

Ca^2+^ has been demonstrated to be a key player in pancreatic (Ren *et al*. 2022) functions (Cui and Kanno 1997). Abnormal insulin secretion of pancreas β cells is a major defect in type 2 diabetes. Impaired mitochondrial function and abnormal Ca^2+^ absorption have been previously found in studies in human and animal diabetic models. NMT experiments found that the change in Ca^2+^ influx caused by the abnormal mitochondria of pancreatic beta cells rather than Ca^2+^ channel activity is the main reason for abnormal insulin secretion in diabetic mice (Li *et al*. 2012a). Islet β cell transplantation is an ideal treatment for type-I diabetes and generating β cells from induced pluripotent stem cells (iPSCs) from patients is a promising strategy. Increased glucose levels lead to membrane depolarization resulting in an influx of Ca^2+^ which triggers insulin exocytosis. Therefore, researchers used Ca^2+^ influxes as one of the benchmarks to evaluate whether the pancreas β cells derived from iPSCs are healthy (Bai *et al*. 2021).

NMT also was widely applied in pharmacological research. Bumetanide is a potent loop diuretic increasing the urine flow by blocking NKCC2, which may relate to its side effects in the digestive system, such as loss of appetite, nausea or/and vomiting due to abnormal H^+^ secretion. H^+^ fluxes in the gastric mucosa of mice revealed by NMT demonstrate that bumetanide (10 μmo/L) significantly inhibited histamine-induced H^+^ fluxes, but had no effect on basal H^+^ fluxes, suggesting NKCC plays a role in acid secretion (Zheng *et al*. 2020). Entacapone, an inhibitor of Catechol-O-methyltransferase (COMT), is widely used in the treatment of Parkinson's disease (PD). However, about 10% of PD patients with entacapone treatment clinically experience diarrhea. NMT results indicate that the cause of the gastrointestinal side effects of entacapone is primarily the profound secretion of Cl^−^ (Li *et al*. 2015). Emodin (1,3,8-trihydroxy-6-methylanthraquinone) is a well-known ingredient of herb-based laxatives, but the working mechanism is still unclear. Cl^−^ secretion was found to be the main player in the emodin-induced colonic physiological process accompanied by mast cell degranulation and activation of cholinergic and non-cholinergic submucosal neurons (Xu *et al*. 2012).

NMT has also been used to detect apoptosis, which often occurs in inflammatory responses. Similar to Ca^2+^, which has been demonstrated to be involved in ROS production and mitochondria depolarization (Hu *et al*. 2008), K^+^ has been proven to be a player in apoptosis. Programmed cell death (apoptosis) occurs in almost all cells, including oocytes and embryos. Understanding the mechanism of apoptosis can provide cues for preterm birth, cancer and aging. When cells initiate programmed death by H_2_O_2_, cells shrink, potassium channels are activated, and K^+^ effluxes are detected outside the embryos (Trimarchi *et al*. 2000a, b).

Alzheimer's disease (AD) is a common, irreversible central nervous system degenerative disorder characterized by progressive cognitive dysfunction, memory loss, and psychobehavioral changes. Its pathology is characterized by massive extracellular deposition of amyloid-β (Aβ) that forms senile plaques and the intracellular accumulation of abnormally modified tau protein that forms neurofibrillary tangles (NFTs). The main pathogenesis hypotheses include: Aβ tangles, Tau hyperphosphorylation, energy metabolism disorders, oxidative stress, excitatory neurotoxicity, metal ion homeostasis, *etc*. (Chung *et al*. 2010; Ray *et al*. 2011; Shabala *et al*. 2010). Among them, the changes of Ca^2+^, K^+^, H^+^, O_2_, and H_2_O_2_ plasma dynamic balance are closely related to the development of AD, which could be detected using NMT. Evidence suggest that transmembrane Ca^2+^ transportation regulation is crucial to understand the mechanism of many human diseases (Li *et al*. 2011). Especially, the intracellular Ca^2+^ overload is an important mechanism for the cytotoxic effects of Aβ. Therefore, rapid and accurate detection of dynamic changes in neuronal transmembrane Ca^2+^ flux can not only help to understand the mechanism of cell maintenance of Ca^2+^ homeostasis and normal functional activities, but also help to reveal the pathogenesis of AD and other diseases related to Ca^2+^ signaling. Researchers used NMT to study the effect of a novel anti-T2DM drug GLP-1/GIP/Gcg tri-receptor agonist (Triagonist) on the transmembrane fluxes of Ca^2+^ in neurons in *ex vivo* brain slices. The results showed that Triagonist maintained the Ca^2+^ homeostasis of neurons and avoided intracellular Ca^2+^ overload by regulating the transmembrane Ca^2+^ fluxes of neurons in the CA1 region of the hippocampus of 3xTg-AD mice (Li *et al*. 2020). Moreover, both K^+^ and Ca^2+^ have been found to be key players in the immune system and the brain (Shu *et al*. 2007). It is imperative to correlate the deposition of amyloid-β (Aβ) that forms senile plaques and the homeostasis of K^+^ and Ca^2+^ to tackle the mechanism of how AD develops. K^+^/Ca^2+^ fluxes data also suggest that long term exposure to Aβ is detrimental since it diminishes the ability of cortical neurons to maintain K^+^/Ca^2+^ homeostasis, which in turn may provide a new insight into the early indicators of AD (Ray *et al*. 2011; Shabala *et al*. 2010). One well-known feature of AD is a relatively higher level of reactive oxygen species (ROS) that overcomes the anti-oxidative stress mechanism of the brain. Meanwhile, the Cu (II)–Aβ complex has been demonstrated to be highly toxic to neurons, and Zn7MT-2A can alleviate the toxicity. In a previous study, a significant K^+^ efflux was detected with the presence of Cu(II)-Aβ1-40 suggesting that Cu(II)–Aβ can lead to disruption of K^+^ homeostasis in cultured cortical neurons, while the addition of Zn7MT-2A can inhibit the disruption (Chung *et al*. 2010; Howells *et al*. 2012).

#### Toxicology

Zebrafish embryos were used as a model to investigate the toxic effects of CuNPs. The results show that CuNP exposure can impair two subtypes of ionocytes and their associated functions, including Na^+^/Ca^2+^ uptake and H^+^/NH_4_^+^ excretion in zebrafish embryos (Lee *et al*. 2020; Shabala *et al*. 2012).

#### Clinical medicine

Cell volume regulation is the basis of various cellular functions, such as cell division, cell proliferation, apoptosis, cell migration, and cell regulation, all of which require changes in cell volume and the involvement of transmembrane ions and molecules (Chen *et al*. 2011). Previously, K^+^ and Cl^−^ were found to be tightly coupled in regulatory volume reduction (RVD), it has been proved, however, not the case by using NMT. Not only for the first time that the transport of K^+^ and Cl^−^ is not coupled during hypotonic-induced RVD, but also H^+^ efflux has been found to play an important role in cell volume regulation, which may be a target for the treatment of nasopharyngeal carcinoma (Yang *et al*. 2012).

Myopia has been a public health issue because of its increasingly high prevalence worldwide. The occurrence of myopia correlates with both the disturbance of ionic homeostasis and the change in the local microenvironment of the ciliary muscle. K^+^ fluxes in ciliary muscles from guinea pigs demonstrated myopia-triggered K^+^ influx, which leads to the disturbance of the microenvironment within ciliary muscles, providing a possible approach to treating myopia in clinical practice (Wu *et al*. 2020). Using live catfish retinal level cells, H^+^ fluxes under the stimulation of a variety of substances suggested that the neurotransmitter Glu induced alkalinization at the outer surface of the cell membrane, and the size of the effect of Glu was related to the buffer, especially extracellular Ca^2+^ (Kreitzer *et al*. 2007).

Understanding the ionic homeostasis of wound healing at the single cell level is crucial to decipher the tissue, organ and or disease physiology (Zhang *et al*. 2022a).

Ca^2+^ influxes are required for single cell wound healing while tested with *Xenopus laevis* oocytes (Luxardi *et al*. 2014).

Furthermore, NMT has also been widely used in the mechanism study of traditional Chinese Medicine. Traditional Chinese medicine and leucous, astragalus sugar complex have been proposed to facilitate the rapid repair process of intestinal mucosa via activation signaling pathway of potassium channel mediated by polyamine. K^+^ effluxes were observed with leucocus and astragalus sugar complex treatments promoting the repair of gastrointestinal mucosal injury (Wang *et al*. 2018).

NMT was adopted to study the acupuncture mechanism. The results indicated that dynamic changes of Ca^2+^, Na^+^, and H_2_O_2_ fluxes had been observed. (1) In the early phase of skeletal muscle regeneration, Ca^2+^ efflux decreased, while Na^+^ influx increased, accompanied by increased H_2_O_2_ efflux; (2) Acupuncture intervention increased Ca^2+^ efflux in the early phase of skeletal muscle regeneration and advanced the Na^+^ influx phase, with the decrease of H_2_O_2_ efflux, and the effect was related to the interaction of TRP with NOX2 (Liu *et al*. 2018).

Membrane depolarization has been found to be accompanied by the transmural pressure, the typical myogenic response of renal and cerebral arteries of the rat. A Cl^−^ efflux from rat cerebral arteries with a temperature dependence correlated with myogenic contraction suggesting that Cl^−^ efflux through Cl^−^ channels contributes to the depolarization associated with myogenic contraction (Doughty and Langton 2001).

Oseltamivir phosphate (OP) is an antiviral drug that is used in the treatment and prophylaxis of both influenza A and influenza B. It is effective against all known influenza viruses that can infect humans, including pandemic influenza viruses and may be the most appropriate antiviral option against avian influenza caused by H5N1. An OP sensor based on ion association complexes and electrochemical reactions using a polyvinyl chloride (PVC) membrane has been successfully developed. The sensor can be used to determine (OP) in tablets. Compared to the reported HPLC method, the developed method is simple, accurate and precise (Hamza *et al*. 2017).

Acupuncture has a good therapeutic effect on skeletal muscle injury, and according to recent research trends, the mechanism is related to the TRP channel-mediated reactive oxygen species signaling pathway to restore early cytoplasmic Ca^2+^ homeostasis. The changes of Ca^2+^, Na^+^, and H_2_O_2_ dynamic flow rates in the early phase after skeletal muscle injury were detected by acupuncture intervention, aiming to explore the role of the interaction between TRP channel and NADPH oxidase 2 in the mechanism of acupuncture effect (Liu and Zhang 2018).

Uncoupling protein (UCP) is an inner mitochondrial membrane protein that eliminates transmembrane proton gradients on both sides of the inner mitochondrial membrane, slowing down the oxidative phosphorylation process driven by the proton gradient and hindering the normal production of ATP. Reduced UCP3 in prediabetes and diabetes is associated with insulin resistance, but the function of UCP3 is unclear. The study of the O_2_ fluxes caused by UCP3 demonstrates no significant change in oxygen consumption in the UCP3 super expression group, and the chemical decoupling agent significantly increased oxygen consumption (MacLellan *et al*. 2005).

### Animal study

#### Entomology

In insects, heterodimeric glycoprotein hormones play an important role in myelinization and cuticle hardening during development. With the recent discovery of two novel subunits forming an additional heterodimeric glycoprotein hormone in mammals, similar glycoprotein hormone forming subunits have been identified in insects. Research done by O’Donnell *et al*. suggests that AedaeGAP2/GPB5 can be used to regulate the physiological phenomenon of low Na^+^ and high K^+^ in the process of digestion and absorption of red blood cells by female mosquitoes, and direct evidence of physiological function has been obtained (Nguyen and Donini 2010; Paluzzi *et al*. 2014).

#### Animal physiology and behavior

E-C coupling contraction of the muscle relies on the movement of Ca^2+^ from different sources, such as the intracellular stores and/or from the extracellular media. The Ca^2+^ flux data suggested that the acetylcholine-induced Ca^2+^ efflux was the result of, first, Ca^2+^ influx through voltage-sensitive L-type Ca^2+^ channels, then the rapid extrusion of Ca^2+^ by an outwardly directed carrier such as the Na-Ca exchanger as demonstrated by Li^+^ substitution experiments. NMT has provided new insights into the active and complex role the sarcolemma plays in Ca^2+^ homeostasis and regulating Ca^2+^ redistribution during excitation-contraction coupling (Devlin and Smith 1996).

### Plants biology

#### Abiotic stress

Quantitative K^+^/H^+^ fluxes under salt stress detected by NMT suggested a K^+^ preservation anti-salt stress mechanism via repolarization of the plasma membrane and silence of Guard cell outward-rectifying K^+^ (GORK) channel (Gong *et al*. 2023; Wu *et al*. 2024; Zeng *et al*. 2024). The other way to control K^+^ fluxes is to go through the key signal ROS that activates the Non-selective cation channel (NSCC) and the pathways generated extracellular of ROS (Pang *et al*. 2006; Shabala and Hariadi 2005; Sun *et al*. 2009c; Tang *et al*. 2018). Taking advantage of direct physiological evidences from NMT, a number of attempts have been made to find out a way to maintain IMH, Na^+^/K^+^ homeostasis specifically, to combat with plant salt stress (Chinnusamy *et al*. 2004; Guo *et al*. 2009; Lou *et al*. 2020; Qu *et al*. 2021; Shabala *et al*. 2005; Shahzad *et al*. 2022; Zhang *et al*. 2022b).

Extreme temperature is a key factor limiting global crop plant distribution and yield. NMT was able to demonstrate that there was a significant difference in Ca^2+^ influxes after cold treatment between COLD1/transgenic lines and corresponding wild types (Ma *et al*. 2015). Supported by Ca^2+^, H^+^, K^+^, and Na^+^ fluxes data, Lsi1 and OsCNGC9 genes have been found to enhance the cold tolerance of crops by regulating low-temperature-induced calcium influx and cytosolic calcium elevation (Liu *et al*. 2024; Wang *et al*. 2021).

Cd^2+^/Cu^2+^/Pb^2+^ influxes have been detected in live samples, such as roots, leaves, algae, biofilms, bacteria, and fungi, as well as with different live parts/organs, such as root xylem, stem, xylem duct, mesophyll cells, intact vacuoles, *etc*. (Cheng *et al*. 2023; Cuin and Shabala 2007; Jiang *et al*. 2022; Li *et al*. 2016, 2017a, 2024; Lv *et* *al*. 2018; Papoyan *et al*. 2007; Yang *et al*. 2022b; Zhang *et al*. 2020). The changes in the absorption of Mg^2+^, K^+^, and Ca^2+^ were studied with plant tissues and cells in the presence of heavy metals (Li *et al*. 2017a, 2017b, 2012b; Lin *et al*. 2013; Ma *et al*. 2016; Wang *et al*. 2022, 2023a). NMT data of Cu^2+^ and Cd^2+^ fluxes help to identify OsHIPP9/BcHIPP16 membrane proteins to absorb nutrient metal Cu and heavy metal Cd in plants (Niu *et al*. 2021; Xiong *et al*. 2023).

Drought is a great threat to crop yields and quality worldwide. lows of H^+^, K^+^, Ca^2+^ and H_2_O_2_ have been monitored with live plant samples to correlate energy, nutrient metabolisms, K^+^ homeostasis, signal transduction, ROS response to water control in plants (Chen *et al*. 2005; Li *et al*. 2019; Mak *et al*. 2014; Sun *et al*. 2024). NMT study provides important evidence for the dual role of plasma membrane H^+^-ATPases in light-induced stomatal opening and ABA-induced stomatal closure, highlighting the importance of H^+^ in plasma membrane cation and anion influx and efflux (Pei *et al*. 2022).

#### Plant immunity

NMT results found that herbivory pretreatment altered Ca^2+^ flux via different Ca^2+^ channels or transporter which help *Ammopiptanthus nanus* to prevent K^+^ leakage (Chen *et al*. 2020). Ca^2+^ flux data also indicated that the wheat susceptibility factor TaBln1, which interacts with TaCaM3 to impair Ca^2+^ influx could inhibit plant defenses (Guo *et al*. 2022). Ca^2+^ influx and H^+^ efflux data indicated that eATP-promoted stomatal opening possibly involves the heterotrimeric G protein, ROS, cytosolic Ca^2+^, and plasma membrane H^+^-ATPase (Gong *et al*. 2023; Hao *et al*. 2012; Sun *et al*. 2012; Zhao *et al*. 2016). NMT results indicated that melatonin, H_2_O_2_ and Ca^2+^ attenuated ABA‐induced K^+^ efflux and subsequent cell death (Guo *et* *al*. 2023).

#### Plant hormones signaling

After Indole acetic acid(IAA) treatment, the H^+^, K^+^, and Ca^2+^ fluxes of *Arabidopsis thaliana* roots were detected (Yang *et al*. 2022a), which provided key evidences for IAA-induced H^+^ alkalinization and then H^+^-induced exosomes in roots (Li *et al*. 2021). NMT experiments have indicated that the Ca^2+^ uptake of the mutant is impaired resulting in the pollen tube tip not moving towards the ovule. Meanwhile, the fluxes of Cl^−^ and K^+^ did not differ significantly between mutant and wild type (Meng *et al*. 2020).

#### Plant nutrition

Sa *et al*. (2019) found that root-associated ectomycorrhizal fungi (EMF) are involved in promoting root NO_3_^−^ uptake of poplar by detecting NO_3_^−^ fluxes around the root surface. Similarly, Peng *et al*. (2021) used the NMT to monitor and compare K^+^ flux profiles across the meristematic zone, root elongation and mature zones of *Liquidambar styraciflua* and observed that more K^+^ is absorbed by the roots colonized by a common soil saprotroph, *Clitopilus hobsonii*. Sun *et al*. (2022) demonstrated that fungus-regulated NO_3_^−^/NH_4_^+^ dynamics alters plant response to NO_3_^−^/NH_4_^+^ nutrition during symbiotic fungus *Phomopsis liquidambaris*-*Arabidopsis* interaction by examining N fluxes at the plant-fungal interface (Peng *et al*. 2021; Sa *et al*. 2019; Sun *et al*. 2022).

The acidity and alkalinity of the surrounding environment also affect ion exchange. In soybeans and coniferous trees, proton efflux at lower pH is beneficial for maintaining sustained absorption of NH_4_^+^, for plants to adapt to acidic soil (Hawkins and Robbins 2010). *Glomus mosseae* (GM, fungus) and *Bradyrhizobium japonicum* (BJ, fungus) promoted the outflow of H^+^ in soybean roots, and the increase of phosphorus content was positively correlated with the outflow of H^+^ (Ding *et al*. 2012).

### Environmental science

Bacteria biofilms use potassium channel-mediated electrical signals for cell-to-cell communication although it is unknown whether these signals play a role in *Geobacter sp.* when surrounded by an intense electric field.

Steady and reliable K^+^ fluxes detected by NMT provide preliminary evidence to reveal the role of potassium channels in electroactive biofilms in *Agrobacterium* reducing ensembles (Jing *et al*. 2020).

Seagrass is the only higher angiosperm that submerges in seawater. The seagrass ecosystem is, nevertheless, subject to numerous stressors brought on by human activity, including hypoxia, hyper salinity, eutrophication, temperature, and others. The findings from NH_4_^+^ and H^+^ flux data demonstrated that an increase in NH_4_^+^ content in seawater encouraged absorption, which might reprogram the nitrogen metabolism (Chen *et al*. 2019; Fang *et al*. 2020).

The safety of freshwater ecosystems is critical to humans, so real-time monitoring of levels of harmful chemicals and biological agents in freshwater environments is essential. Using O_2_ fluxes as a Biosafety Indicator, a real-time effect of atrazine, cadmium chloride, pentachlorophenol, malathion, and potassium cyanide on respiratory oxygen consumption in fathead minnow embryos two days after fertilization was detected (Sanchez *et al*. 2008).

Plants use environmental signals to guide root growth and response so that they can adapt to the environment. For instance, roots can direct their growth and response in relation to gravity, light, gradients of temperature, humidity, ions, chemicals and oxygen. These environmental signals will be translated into physiological responses via ionic/molecular transportation. In root gravitropism, H^+^ efflux or Ca^2+^ influx in root apexes was weaker in IPG-grown roots than those in TPG-grown roots (Xu *et* *al*. 2013).

A list of live samples tested by NMT is provided in Table 1, and a list of categorized references by NMT applications fields is also made available as a quick reference with NMT (supplementary Table S1).

**Table 1:** Summary of samples tested by NMT

Plant samples	Number of publications
*Arabidopsis thaliana*	107
Rice	79
Poplar	38
Wheat	36
Tobacco	24
Apple	14
Cotton	12
Tea plant	11
Corn	10
Alfalfa, rape	9
Soybean	8
Sweet potato	7
Tomato, cucumber, southeast *Sedum*, mining accompanying *Sedum*	6
Trifoliate orange, Spruce	5
*Solanum nigrum*, *Limonium bicolor*, duckweed, mangrove, *Leymus chinensis*	4
Grape, *Porphyra haitanensis*, Chinese cabbage, *Suaeda salsa*, pumpkin, white thorn, cassava, *Phyllostachys edulis*	3
Potato, autumn eggplant, Peas, pear, star grass, ryegrass, *Polyporus umbellatus*, mung bean, maple fragrance, sweet sorghum, willow	2
Candlestick fruit, tall fescue, white stem, green cottage, *Phellodendron amurense*, licorice, lily, Bidong eggplant (*Petunia*), peanut, Chinese fir, Chinese cabbage, *Spartina alterniflora*, Jiangli, child ginseng, reed, Chinese chestnut, watermelon, *Zizania latifolia*, Minjiang fir, red thick shell (begonia fruit), *canna* plantain, Xiaojin Haitang, Heguo taro, mulberry, sand holly, water caltrop, black fruit goji berries, *Halophila beccarii*, sugarbeet, banana, *Malus hupehensis*, Mi Zi, *Dendrobium officinale*, strawberry, Huanghua Hongsha, white feather fan bean, cucumber grass, bitter grass, *Microsorum pteropus*, goldfish grass, *Sorghum*, millet, blueberries, lettuce, Ningxia goji berries, *Spiraea japonica*, sour jujube, oats, *Bidens* bipinnata, *Iris*, *Citrus*, wolf tail grass, *Celosia argentea*	1
Other samples	
Zebrafish	9
Green guppy	6
Clawed toad	4
Yeast cell	4
Metallic material	3
Fungi	3
Biological materials, rat colon, *Phanerochaete chrysosporium*, mice	2
Rat, guinea pig, sludge, mouse pancreatic islet tissue, nasopharyngeal cancer cells, human oral epidermoid carcinoma cells, C6 glioma cells, oral squamous cell carcinoma cells, pearl oyster, liver cells, acellular cortical bone slices, I- β cell, *Escherichia coli*, *Aspergillus niger*, *Geobacter*, microalgae	1

## CONCLUSION

Abnormal IMHs can lead to pathology, while the activities of ions and small molecules are relatively difficult to study.

NMT, an integration of multi-techniques and multi-disciplinary knowledge, is evolving. It is constantly being improved functions to better fit their specific studies (Chi *et al*. 2021; Li *et* *al*. 2014; Lv *et al*. 2013; Song *et al*. 2017; Zhu *et al*. 2007).

The involvements of H^+^ and O_2_ in tumor physiology have been studied (Hsu and Sabatini 2008; Parks *et al*. 2013; Seyfried and Shelton 2010), as well as H^+^ and/or O_2_ manipulations in tumor micro-environments (Bailey *et al*. 2012; Parks *et al*. 2013; Yang and Li 2019; Zhang *et* *al*. 2021).

A few decades have passed since the first Ca^2+^ flux data was collected by biologists from a single cell (Jaffe and Nuccitelli 1974; Kuhtreiber and Jaffe 1990). NMT-based ions and molecules Omics (imOmics) is the study of IMH pertinent to a specific physiological process via collections and integral analysis of ions and/or molecules activities (Xu *et al*. 2023).

In this multi-dimensional-omics age, scientists are trying to make a functional network map, it has been demonstrated that the study of IMHs by NMT could play a significant role in not only connecting but verifying data from other techniques for a better understanding of a specific physiological function, more importantly from live samples.

## Conflict of interest

Kai Sun, Yunqi Liu, Yanshu Pan, Dongwei Di, Jianfang Li, Feiyun Xu, Li Li, Yoshiharu Mimata, Yingying Chen, Lixia Xie, Siqi Wang, Wenqian Qi, Yan Tang, Huachun Sheng, Bing Wang, Ruixue Sun, Dingquan Tan, Daohong Fu, Ye Yin, Ao Xue, Yichao Shi, Wenjing Shao, Lei Gong, Zhijian Jiang, Wei Zhang, Qiangsheng Wu, Yaosheng Wang, Minglin Lang, Wenxiu Ye, Weifeng Xu, Shuhe Wei, Weiming Shi and Yue Jeff Xu declare that they have no conflict of interest.
