# Correction to: Non-invasive micro-test technology and applications

**DOI:** 10.52601/bpr.2025.250901

**Published:** 2025-06-30

This is a correction to: Kai Sun，Yunqi Liu，Yanshu Pan，Dongwei Di，Jianfang Li，Feiyun Xu，Li Li，Yoshiharu Mimata，Yingying Chen，Lixia Xie，Siqi Wang，Wenqian Qi，Yan Tang，Huachun Sheng，Bing Wang，Ruixue Sun，Dingquan Tan，Daohong Fu，Ye Yin，Ao Xueao Shi，Wenjing Shao，Lei Gong，Zhijian Jiang，Wei Zhang，Qiangsheng Wu，Yaosheng Wang，Minglin Lang，Wenxiu Ye，Weifeng Xu，Shuhe Wei，Weiming Shi，Yue Jeff Xu (2025) Non-invasive micro-test technology and applications. Biophysics Reports 11(2): 96-111. https://doi.org/10.52601/bpr.2024.240009.

In the originally published version of this article, there were several typographical errors in Figure 1. The corrected version of Figure 1 is provided here for reference.

**Figure 1:**
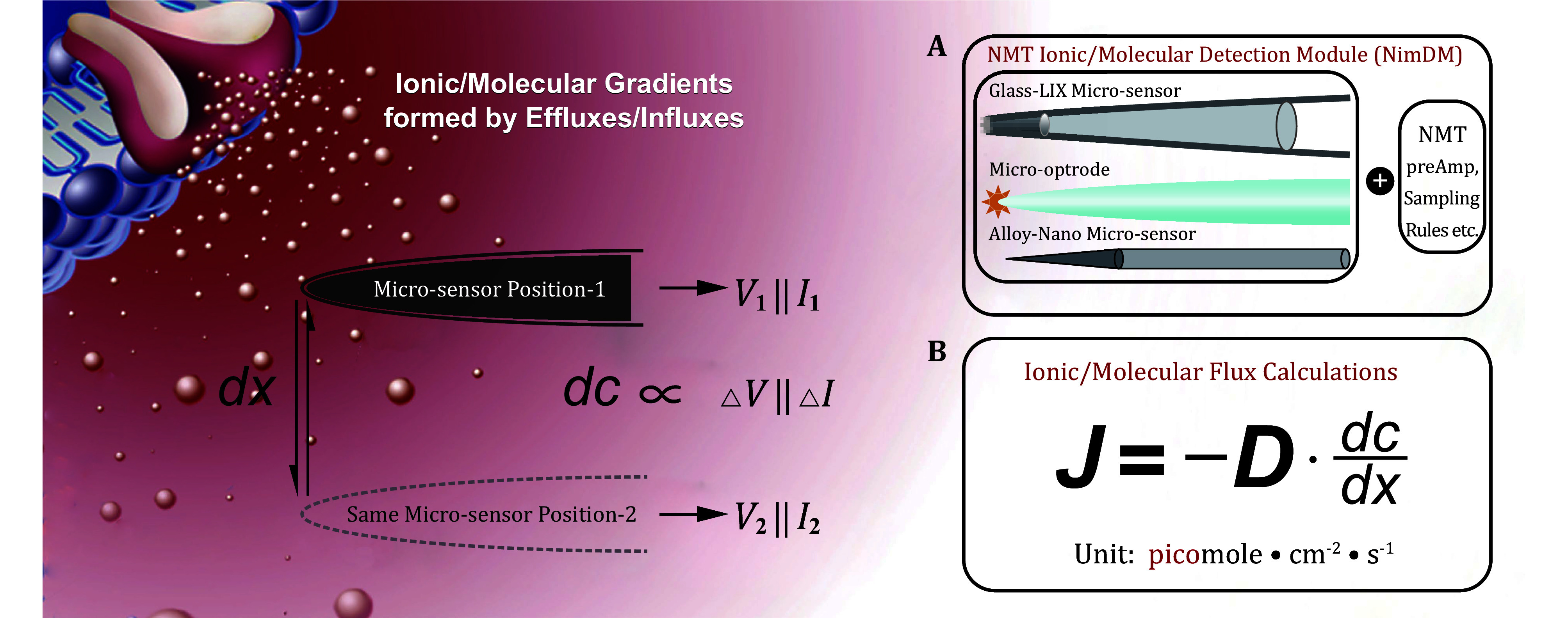


## Conflict of interest

 declare that they have no conflict of interest.

